# The anticancer and antibacterial potential of bioactive secondary metabolites derived From bacterial endophytes in association with *Artemisia absinthium*

**DOI:** 10.1038/s41598-023-45910-w

**Published:** 2023-10-27

**Authors:** Mohammad Sadegh Damavandi, Hasan Shojaei, Bahram Nasr Esfahani

**Affiliations:** Department of Microbiology, School of Medicine, Isfahan University of Medical Sciences, Isfahan, Iran

**Keywords:** Cancer therapy, Antimicrobials, Applied microbiology, Phenotypic screening

## Abstract

The continuous search for secondary metabolites in microorganisms isolated from untapped reservoirs is an effective prospective approach to drug discovery. In this study, an in-depth analysis was conducted to investigate the diversity of culturable bacterial endophytes present in the medicinal plant *A. absinthium*, as well as the antibacterial and anticancer potential of their bioactive secondary metabolites. The endophytic bacteria recovered from *A. absinthium*, were characterized via the implementation of suitable biochemical and molecular analyses. Agar well diffusion and broth microdilution were used to screen antibacterial activity. SEM was performed to assess the impact of the extracted metabolite on MRSA strain cell morphology. Apoptosis and cytotoxicity assays were used to evaluate anticancer activity against MCF7 and A549. The FTIR, GC–MS were used to detect bioactive compounds in the active solvent fraction. Of the various endophytic bacteria studied, *P. aeruginosa* SD01 showed discernible activity against both bacterial pathogens and malignancies. The crude ethyl acetate extract of *P. aeruginosa* SD01 showed MICs of 32 and 128 µg/mL for *S. aureus* and MRSA, respectively. SEM examination demonstrated MRSA bacterial cell lysis, hole development, and intracellular leaking. This study revealed that the crude bioactive secondary metabolite SD01 has potent anticancer activity. In this study, 2-aminoacetophenone, 1,2-apyrazine-1,4-dione, phenazine and 2-phenyl-4-cyanopyridine were the major bioactive secondary metabolites. In conclusion, our findings indicate that the bacteria recovered from *A. absinthium* plants and in particular, *P. aeruginosa* SD01 is a remarkable source of untapped therapeutic, i.e., antimicrobial and anticancer compounds.

## Introduction

In recent years, a significant concern for the healthcare system is the rise of antimicrobial and chemotherapeutic drugs resistance. Therefore, there is an urgent need to discover and develop novel anticancer and antibiotics^1^. Consequently, a resurgence of interest has arisen regarding the exploration of microorganisms as a source for novel leads, predicated on the impressive achievements of microbial metabolites as the foundation for the development of efficacious antibiotics, antineoplastic agents, and agricultural chemicals^2^. In this particular context, microbial symbioses hold a substantial importance. This is primarily due to the growing body of evidence that recommends the involvement of microbial bioactive compounds, similar to those that are utilized as antibiotics, in both microbe-microbe as well as microbe-host communication mechanisms^3^.

Bacterial endophytes may be characterized as microorganisms that inhabit the interior plant tissues without manifesting any outward indications of infection or adverse impact on their host^4^. There is compelling evidence to support the notion that endophytes possess advantageous attributes for their respective host plants, including facilitating growth, providing insect and pest resistance, exerting antimicrobial effects against plant pathogens, and offering stress management capabilities^5^. There has been a growing interest in the investigation of endophytic bioactive secondary metabolites due to their multifarious structural categories and diverse bioactive properties^6^. As of current, a limited number of plants have been subject to investigation regarding their endophytic biodiversity and their capacity to generate bioactive secondary metabolites. Endophytes are presently acknowledged as an exceptional repository of bioactive natural products owing to their occupancy of a plethora of distinctive biological niches, ranging from various atypical habitats, thereby amounting to millions of such niches^7^.

Virtually, all the medicinal plant species on earth are the hosts of one or more types of endophytic bacteria. Endophytes can co-evolve with plant hosts and undergo species-specific interactions. Medicinal plants i.e., Artemisia are potential hosts to surplus advantageous endophytes. *Artemisia* is a genus of plants highly valued as a source of metabolites useful in, for example, medicine and biopesticides^8,9^.

Among the examined flora, *Artemisia absinthium* (*A. absinthium*) holds a significant position as a medicinal plant in the annals of medical practices across Europe, West Asia, and North America, and it is employed in both allopathic and homeopathic methodologies. The biological activity of this species is attributed to a range of raw materials including essential oil, bitter sesquiterpenoid lactones, flavonoids, other bitterness-imparting compounds, azulenes, phenolic acids, tannins and lignans, all of which have been confirmed through scientific research. These materials exhibit a range of noteworthy biological properties, including antiprotozoal, antibacterial, antifungal, anti-ulcer, hepatoprotective, anti-inflammatory, immunomodulatory, cytotoxic, analgesic, neuroprotective, anti-depressant, procognitive, neurotrophic, and cell membrane stabilizing and antioxidant effects^10,11^.

Within the context of Iran, it has been reported that medicinal plants serve as a host for certain bacterial endophytes that play a role in the co-production of biologically active secondary metabolites^12^. Our country possesses an unparalleled assemblage of plant diversity, distinguishing it from other countries in the Middle Eastern region. The comprehensive range of flora within a given ecosystem engenders a corresponding assemblage of endophytic bacteria, each of which exhibits unique capabilities in synthesizing bioactive secondary metabolites.

The objective of the present investigation was to conduct a screening, identification, and characterization of the potential bioactive secondary metabolites implicated in bacterial endophytes, which represent a significant source of distinct bioactive secondary metabolites derived from medicinal plants indigenous to Iran.

## Results

### Identification of bacterial endophytes

**Figure 1:**
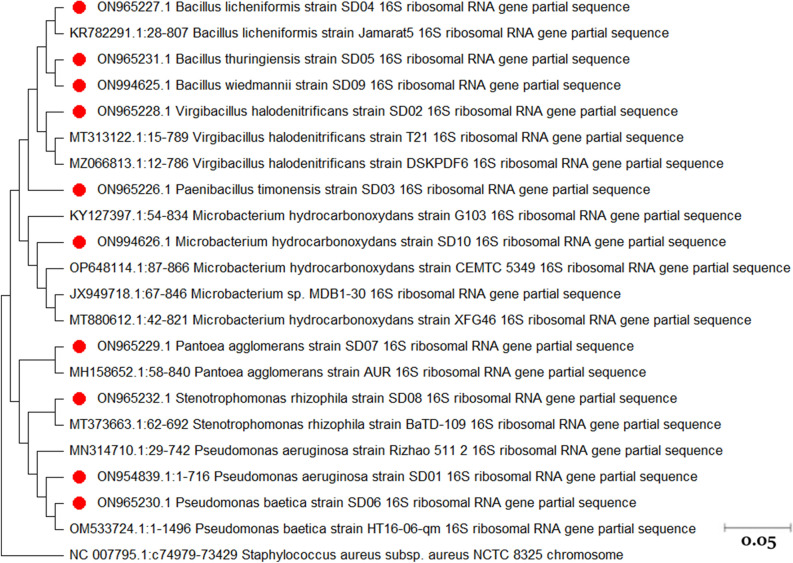
Phylogenetic tree of bacterial endophytes isolated from *A. absinthium* and their closest relatives from GenBank. The evolutionary history was inferred using the Neighbor-Joining method^53^. The optimal tree is shown. The tree is drawn to scale, with branch lengths in the same units as those of the evolutionary distances used to infer the phylogenetic tree. The evolutionary distances were computed using the Maximum Composite Likelihood method^54^ and are in the units of the number of base substitutions per site. This analysis involved 22 nucleotide sequences. All ambiguous positions were removed for each sequence pair (pairwise deletion option). There were a total of 1874 positions in the final dataset. Evolutionary analyses were conducted in MEGA11^51^.

A total of 10 bacterial endophytes have been isolated from *A. absinthium*, representing seven genera and ten species (Table 1). Among the all isolates, 60% (6/10) were Gram positive and 40% (4/10) were Gram negative bacteria. Endophytic bacteria belonging to the *Bacillus* genus were the most abundant bacteria isolated from *A. absinthium*. The phylogenetic tree presented in Fig. 1 illustrates the relationship between bacterial endophytes obtained from *A. absinthium* and their most closely related counterparts from the GenBank database.

**Table 1:** Biochemical characteristics and molecular identification of *A. absinthium* bacterial endophytes.

Isolate ID	Morphology	Endospore	Catalase	Oxidase	Gelatin hydrolysis	Starch hydrolysis	IMViC Tests				Carbohydrate fermentation									ADH	LDC	ODC	H2S	Urea	TDA	ONPG	Nitrate	Motility	*16 s rRNA* Identified Bacteria (accession number)
							Indole	MR	VP	Citrate	Glucose	Arabinose	Raffinose	Sorbitol	Rhamnose	Sucrose	Xylose	Lactose	Dextrose										
SD01	G-, R	−	+	+	+	+	−	−	−	+	−	−	−	−	−	−	−	−	−	+	−	−	−	−	−	−	+	+	*P. aeruginosa (*ON954839*)*
SD02	G + , R	+	+	+	+	−	−	−	+	+	−	−	−	−	−	−	−	−	−	−	−	−	−	+	−	−	−	+	*V. halodenitrificans (*ON965228*)*
SD03	G + , R	+	+	−	−	+	−	−	−	−	+	−	−	−	−	−	+	−	−	−	−	−	−	−	−	−	+	+	*P. timonensis (*ON965226*)*
SD04	G + , R	+	+	+	+	+	−	−	+	+	+	+	−	+	+	+	−	−	+	+	−	−	−	−	−	+	+	+	*B. licheniformis (*ON965227*)*
SD05	G + , R	+	+	−	+	−	−	−	+	−	+	−	−	−	−	+	−	−	+	−	−	−	−	−	−	−	+	+	*B. thuringiensis (*ON965231*)*
SD06	G-, R	−	+	+	+	+	−	−	−	+	−	−	−	−	−	−	−	−	−	+	−	−	−	−	−	−	−	+	*P. baetica (*ON965230*)*
SD07	G-, R	−	+	−	+	−	−	−	+	+	+	+	+	+	+	+	+	−	−	+	−	−	−	−	−	+	+	+	*P. agglomerans (*ON965229*)*
SD08	G-, R	−	+	+	+	−	−	+	−	+	−	−	−	−	−	−	−	−	−	−	−	−	−	−	−	+	+	+	*S. rhizophila (*ON965232*)*
SD09	G + , R	+	+	−	+	+	−	−	+	+	+	−	−	−	−	+	−	−	−	−	−	−	−	−	−	−	+	+	*B. wiedmannii (*ON994625*)*
SD10	G + , R	−		−	−	+	−	+	−	+	−	−	−	−	+	+	+	−	−	−	−	−	−	−	−	−	−	+	*M. hydrocarbonoxydans (*ON994626*)*

G+ : Gram positive, G-: Gram negative, MR: Methyl red, VP: Voges-Proskauer, ADH: Arginine-dihydrolase, LDC: Lysine decarboxylase, ODC: Ornithine decarboxylase, TDA: Tryptophane deaminase, ONPG: O-Nitrophenyl-β-D-galactopyranoside. *Pseudomonas aeruginosa, Virgibacillus halodenitrificans, Paenibacillus timonensis*, *Bacillus licheniformis*, *Bacillus thuringiensis*, *Pseudomonas baetica*, *Pantoea agglomerans*, *Stenotrophomonas rhizophila*, *Bacillus wiedmannii*, *Microbacterium hydrocarbonoxydans.*

### Screening for antimicrobial activity

**Figure 2:**
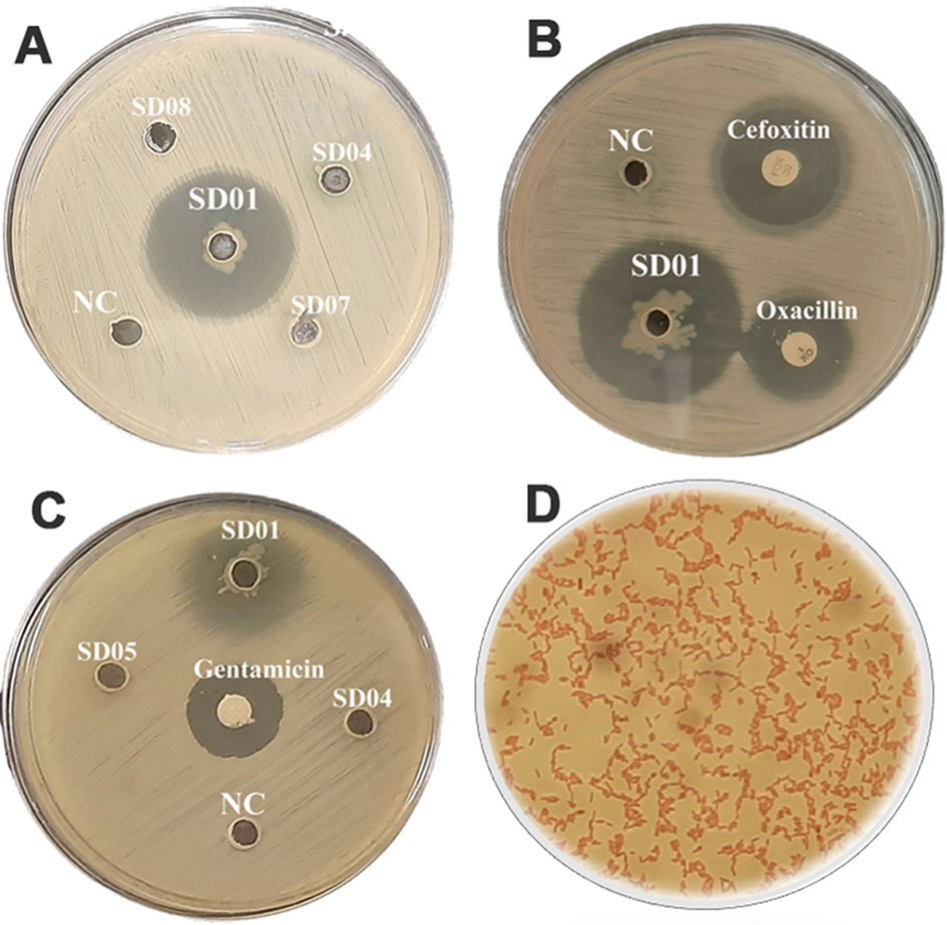
Screening for antibacterial activity by agar well diffusion method. (**A**); *S. aureus* ATCC 25,923, (**B**); MRSA ATCC 33,592, (**C**); *E. coli* ATCC 25,922, NC; Negative control, (**D**); Gram stain of *P. aeruginosa* SD01.

**Figure 3:**
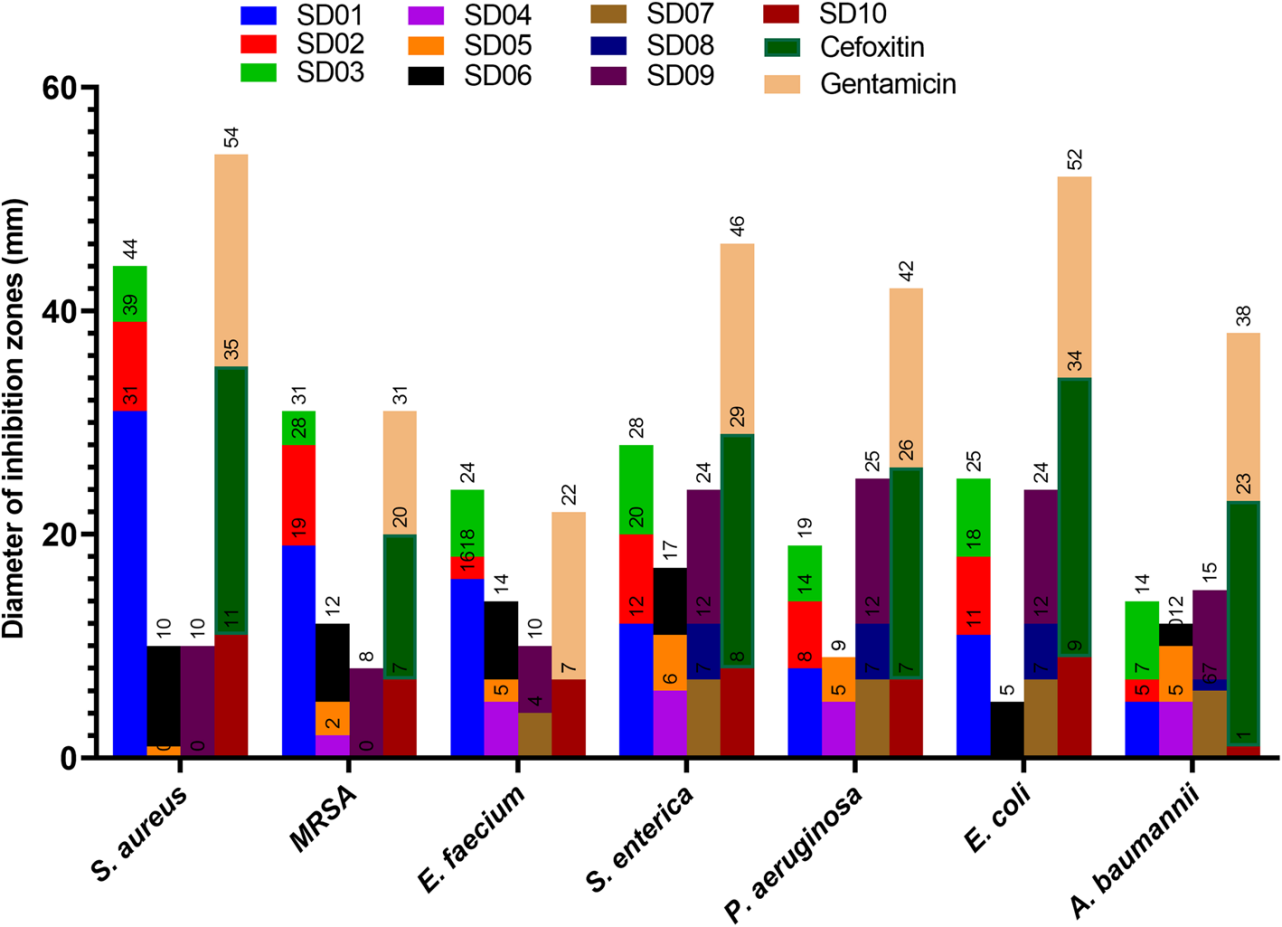
Comparison antimicrobial activity of concentrated crude extracts of secondary metabolites from *A. absinthium* associated bacterial endophytes by agar well diffusion method.

According to the agar well diffusion method (Fig. 2), all of ten endophytic bacteria isolates demonstrated antibacterial activities against at least one pathogenic bacterium and had different inhibition zone ranging from 3 to 31 mm. The most potent antibacterial activity was demonstrated by a *P. aeruginosa* SD01 isolate against *S. aureus* ATCC 25,923 and MRSA ATCC 33,592 with inhibition zone of 31 mm and 19 mm (Fig. 3), respectively. The minimum inhibitory concentration (MIC) values of bioactive secondary metabolites against all pathogen microorganisms are shown in Table 2.

**Table 2:** Antimicrobial activity of concentrated crude extracts of secondary metabolites from bacterial endophytes associated with *A. absinthium*.

SM*/Antibiotic	*S. aureus*^a^	MRSA^b^	*E. faecium*^c^	*S. enterica*^d^	*P. aeruginosa*^e^	*E. coli*^h^	*A. baumannii*^g^
Diameter of inhibition zones (mm)/MIC (μg/mL)*							
SD01	**31/32**	**19/128**	16/256	12/512	8/1024	11/512	5/1024
SD02	8/512	9/1024	2/ND	8/256	6/1024	7/512	2/ND
SD03	5/1024	3/ND	6/ > 2048	8/512	5/1024	7/512	7/ > 2048
SD04	0/ND	2/ND	5/ > 2048	6/2048	5/ > 2048	0/ND	5/ > 2048
SD05	1/ND	3/ND	2/ > ND	5/1024	4/ND	0/ND	5/1024
SD06	9/1024	7/ > 2048	7/ > 2048	6/1024	0/ND	5/1024	2/ND
SD07	0/ND	0/ND	4/ > ND	7/2048	7/ > 2048	7/1024	6/2048
SD08	0/ND	0/ND	0/ND	5/2048	5/ > 2048	5/ > 2048	1/ND
SD09	10/512	8/2048	6/1024	12/512	13/256	12/512	8/1024
SD10	11/256	7/2048	7/ > 2048	8/1024	7/2048	9/1024	1/ND
Cefoxitin	24/1	**13/32**	ND/ND	21/1	19/4	25/1	22/8
Gentamicin	19/2	**11/64**	15/4	17/2	16/4	18/1	15/8

*Extracted Secondary metabolites, a: *Staphylococcus aureus* ATCC 25,923, b: Methicillin-resistant *S. aureus* ATCC 33,592, c: *Enterococcus faecium* ATCC 51,299, d: *Salmonella enterica* ATCC 14,028, e: *Pseudomonas aeruginosa* 27,852, h: *Escherichia coli* ATCC 25,922, g: *Acinetobacter baumannii* ATCC 19,606. *MIC values were determined up to 2048 μg/mL, extracts that did not show inhibition at this higher tested concentration are reported as > 2048 μg/mL, ND: Not determined.

Significant values are in bold.

### Scanning electron microscopic studies

**Figure 4:**
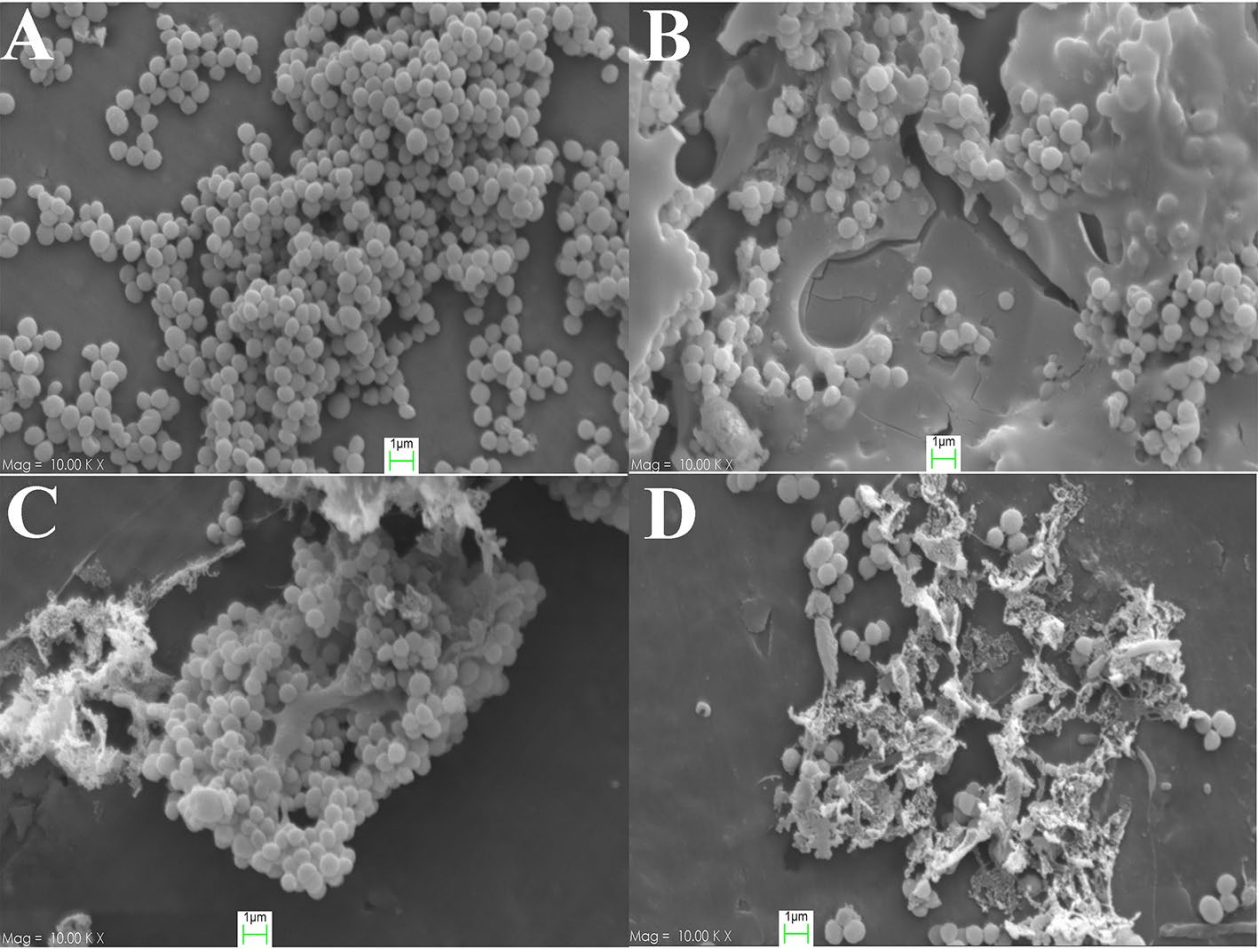
Scanning electron microscopic of bioactive secondary metabolite extracted from SD01 strain. (**A**); *S. aureus* without treatment (morphology control), (**B**); Chlorhexidine gluconate (0.2%) (positive control), (**C**); *S. aureus* treatment with 32 ug/ml of bioactive secondary metabolite extracted from SD01, (**D**); MRSA treatment with 128 ug/ml of bioactive secondary metabolite extracted from SD01.

In the SEM investigation without bioactive secondary metabolite treatment, *S. aureus* displayed regular cell morphology, with uniform sizes and distributions (Fig. 4A). In contrast, bacterial cells that were subjected to chlorhexidine gluconate (Fig. 4B) and the bioactive secondary metabolites of SD01 exhibited surface depressions and biconcave morphologies due to the rupture of their membranes. After 12 h of incubation with the bioactive secondary metabolite (32 µg/mL for *S. aureus* ATCC 25,923 and 128 µg/mL for MRSA ATCC 33,592), the bacterial cells showed signs of lysis and pore formation on the cell surface, as well as leakages of intracellular components (Fig. 4C,D). The occurrence of central depressions or collapses was detected in a substantial proportion of bacterial cells treated with bioactive secondary metabolites. At concentrations equivalent to the MIC, the bioactive secondary metabolite induced clusters of aberrantly shaped bacterial cells exhibiting indications of compromised cellular morphology and leakage of cytoplasmic contents onto the bacterial surface.

### Anticancer and apoptosis evaluation

**Figure 5:**
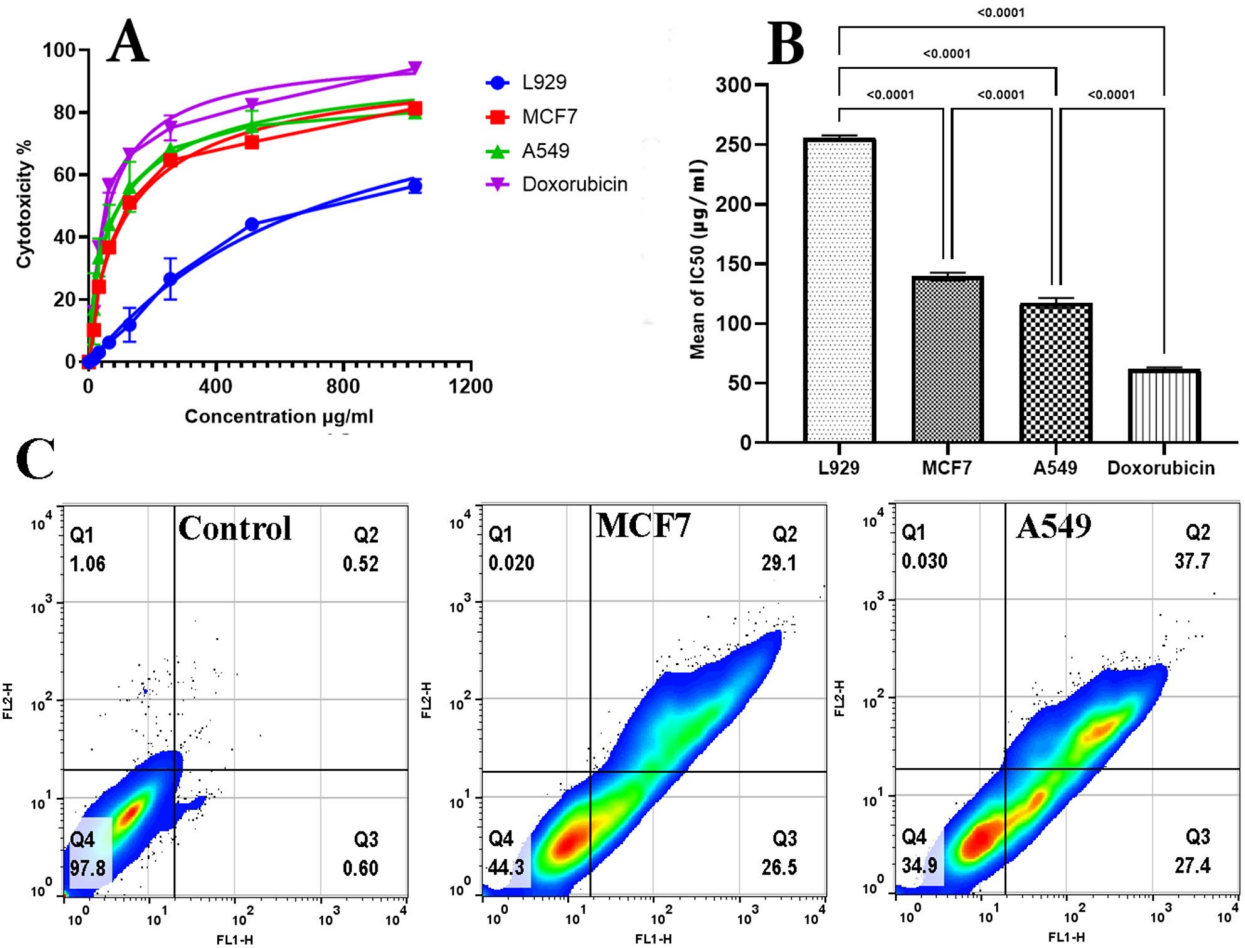
Anticancer and apoptosis evaluation. (**A**); Cytotoxicity assay of bioactive secondary metabolite extracted from SD01 by MTT method, (**B**); Comparison of mean IC50 of bioactive secondary metabolite extracted from SD01 between three cell lines. Doxorubicin was used as the positive control. (**C**); Apoptosis evaluation by Annexin V binding assay. The FSC/SSC plot was used to determine the analysis border in relation to the untreated cells (control).

To evaluate the anticancer activity, it was found that the normal fibroblast cell line L929 was not significantly affected by the cytotoxicity of SD01's crude bioactive secondary metabolites at concentrations of 1–1024 μg/ml (Fig. 5A). The crude bioactive secondary metabolites of SD01 had an IC50 value of 256 µg/ml in the L929 normal fibroblast cell line, which was eight times higher than its MIC (32 µg/ml) against *S. aureus*. In contrast, the findings revealed a proportional relationship between escalating doses of crude bioactive secondary metabolites of SD01 and augmented incidence of cell demise in both MCF-7 and A549 cancer cell lines. The experimental findings revealed a substantial disparity in cell death rates between the lowest (16 µg/ml) and the highest (1024 µg/ml) concentrations, with a notable 81% of cell death observed at the highest concentration, and a mere 10% at the lowest concentration. A cell death rate of about 51% was observed at a concentration of 128 µg/ml, which was deemed the IC50 and used for further analysis (Fig. 5B). Figure 5C depicts the incidence of apoptosis subsequent to exposure to both treated and untreated cells, subsequent to 24 h of exposure to SD01's bioactive secondary metabolites at a concentration of 128 µg/ml. Our findings provide evidence that the administration of 128 µg/ml concentration of bioactive secondary metabolites derived from SD01 resulted in significant induction of apoptosis in MCF7 cells. The data reveal that 55. 6% (Q2 + Q3) and 65. 1% (Q2 + Q3) of MCF7 cells underwent programmed cell death upon exposure to the aforementioned concentration of secondary metabolites. No signs of necrosis were detected in the cell lines scrutinized.

### FTIR Spectrophotometer

**Figure 6:**
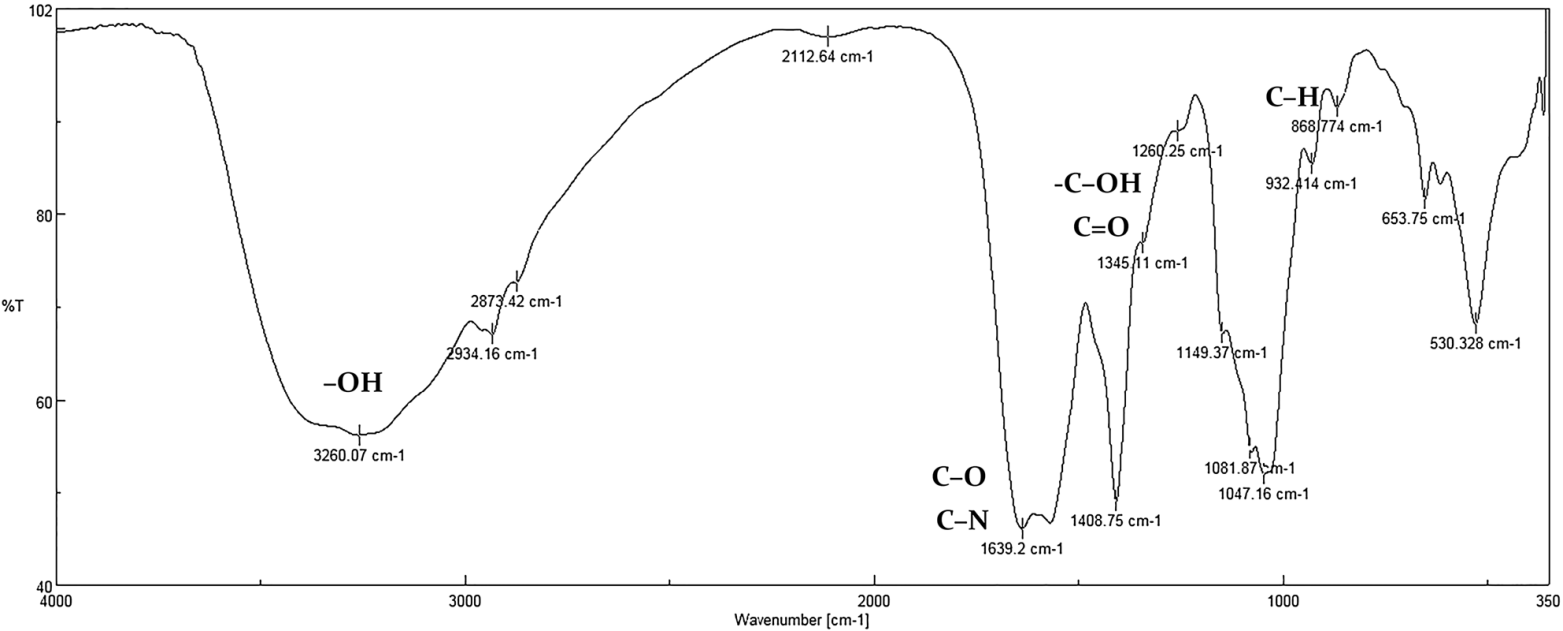
FTIR spectra of bioactive secondary metabolite extracted from SD01.

The possible functional groups of the bioactive secondary metabolites of the bacteria were analyzed using FTIR (Fig. 6). The presence of the hydroxyl and carbonyl groups of flavonoids can be confirmed by vibration spectroscopy techniques^13^, and compared with the peaks that were observed in other studies on flavonoids and antioxidants. The bonds in the region of 802–932 cm-1 can be related to the presence of aromatic C–H groups^14^. The appeared peaks at around 1260 and 1408 cm^-1^ were associated with—C–OH and C=O, respectively^15^ and these peaks are similar to those found for the FTIR spectra of flavonoid quercetin-5′-sulfonated acid^16^. The broad bonds at around 3366, 3382, 3260, and 3379 cm^-1^ were attributed to the stretching vibration of –OH groups which can be associated with alcohols and phenols^17^. However, the OH peak at around 2930, 2928, 2934, and 2929 cm^-1^ usually indicates the presence of water and cannot be applicable for the explanation^14,15^.

The bonds in the range of 500–1500 cm^-1^ represent the carbohydrate fingerprints^18^. Those at 1031, 1049, and 1047 cm^-1^ are corresponding to the stretching vibration of alcoholic groups^19^. The bands in the region of 1600–1700 cm^-1^ were attributed to the bioactive secondary structure of the protein. The peak around 1651 cm^-1^ was associated with amide I (C–O and C–N)^20^. Finally, the peaks presented are similar to the presence of aromatic structures, typical of flavonoids. There are several types of flavonoids, but all share the general structure of C6–C3–C6 phenyl benzopyran, consisting of aromatic rings.

### Detection of bioactive compounds by GC–MS analysis

**Figure 7:**
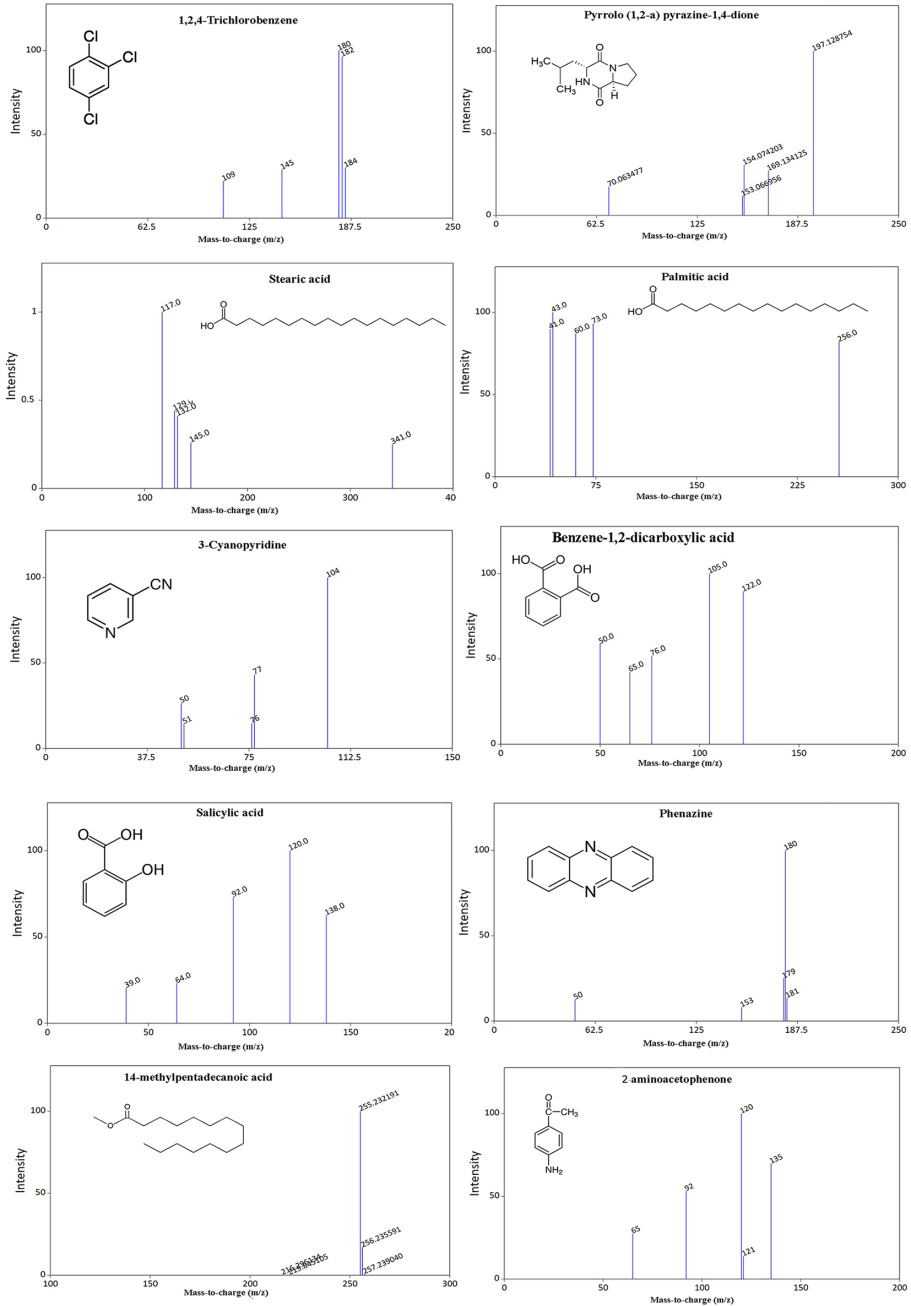
GC–MS spectrum and chemical structures of active secondary metabolites produced by *P. aeruginosa* SD01.

The detection of esters, alcohols, phenols, and cyclic dipeptides in ethyl acetate extract was effectively carried out using GC/MS analysis on complex mixtures. The identification of these chemical compounds was carried out by means of comparing their mass spectra with the database that is currently accessible on the W9N11 MS library. Table 3 illustrates a comprehensive record of the chemical compounds, highlighting their retention time, molecular weight, and molecular formula. Meanwhile, their corresponding chemical structures are represented in Fig. 7.

**Table 3:** List of major compounds identified by GC–MS from secondary metabolites of SD01.

No	RT (min)	Name of compound	Molecular formula	MW	Peak Area %	Qualitative hits from each library %		
						wiley7n.l	NIST05a.L	Pest.1
1	4.562	Salicylic acid	C7H6O3	138.12	3.1	91	87	–
2	5.848	1,2,4-Trichlorobenzene	C6H3Cl3	181.4	0.54	96	95	94
3	7.031	2-aminoacetophenone	C8H9NO	135.16	4.3	94	94	–
4	14.153	14-Methylpentadecanoic acid	C16H32O2	256.42	1.32	72	72	4
5	14.53	n-Hexadecanoic acid	C16H32O2	256.42	8.68	98	83	–
6	16.41	Stearic acid	C18H36O2	284.5	9.39	99	91	–
7	18.255	Pyrrolo (1,2-a) pyrazine-1,4-dione	C10H16N2O2	196.25	32.45	96	96	–
8	18.507	Phenazine	C12H8N2	180.20	9.06	78	78	–
9	18.599	3-Cyanopyridine	C6H4N2	104.11	0.17	70	45	–
10	19.579	Benzene-1,2-dicarboxylic acid	C8H6O4	166.13	59.63	86	86	–

## Discussion

Endophytes are gaining pharmaceutical and industrially relevance as a result of their ability to biosynthesize bioactive secondary metabolites that could serve as biocontrol, antimicrobial, anticancer, and anti-oxidants^21^.

Numerous investigations have been carried out in the last few years concerning endophytes, predominantly in Europe, America, and East Asia that have moderate and humid continental climates as well as tropical rainforests. The endophytic microbiomes' diversity and composition are influenced by ecological features of the host plant and soil, including geographic position, environmental elements, and interactions within the host plant. Most characterized members of bacterial endophytic communities in geographic location from European and East Asia belong to the *Actinobacteria*, *Bacteroidetes*, *Firmicutes*, and *Proteobacteria*^22–25^. In contrast to Europe and East Asia, as well as America, Iran has significant expanses of arid ecosystems, comprising deserts that receive an average yearly rainfall of 134 mm. These deserts are believed to provide a habitat for extreme organisms, including bacterial endophytes^26^. However, as far as our literature review suggests, there have been no reports of bacterial endophytes found in medicinal plants that thrive in the desert regions of Iran. Consequently, the current investigation was carried out to isolate endophytic bacteria and assess their bioactive secondary metabolites from *A. absinthium* cultivated in the arid climate of Iran. We concentrated on the *A. absinthium* plant, as previous studies have demonstrated its exceptional significance in regards to endophytes. Nevertheless, the majority of past investigations have focused mainly on *Artemisia annua*, a medicinal plant whose use has long been reported in China, now naturalized in many other countries in Asia, India, Central and Eastern Europe, in the temperate regions of America, Africa, Australia and in tropical regions^3,7^. It is widely used as a dietary spice, herbal tea, and medicinal plant in the mild climates of Asia, such as China and Korea. That is why we directed our research towards an alternative species, that is, *A. absinthium* that has received less attention. Moreover, almost all studies on *A. absinthium* were conducted in tropical and East Asian regions and have focused on fungal endophytes^8,27–30^. Our study aimed to explore whether this advantageous medicinal plant contains bacterial endophytes and, if confirmed, to describe them.

We were able to isolate ten endophytic bacteria from *A. absinthium*, most of which belonged to *Bacillus* and *Pantoea* bacterial genus. In the investigation conducted by other investigators, the two genera *Bacillus* and *Pantoea* were prevalent among the species other than *A. absinthium* that fall under the *Artemisia* classification^31–33^. In a study conducted by Sebola et al. the prevailing endophytic bacteria isolated from *Crinum macowanii* found to be strains belonging to the *Pseudomonas* genus^34^. The noteworthy aspect of our investigation is that *M. hydrocarbonoxydans*, which is one of the endophytic bacteria obtained from *A. absinthium*, has not been documented as an endophyte before. *M. hydrocarbonoxydans* is crude-oil-degrading Gram-positive bacterium originally isolated from different oil-containing environments^35^. Our research has revealed that this microorganism has the ability to assume the role of an endophyte. Consequently, it can be inferred that vegetation, especially in arid regions that have not previously been investigated for endophytes, may be more likely to contain endophytic bacteria and, therefore, may be considered as potential sources of bioactive secondary metabolites.

The utmost potent among the ten bioactive derivative metabolites attained in this study, was derived from bacterial strains related to the *P. aeruginosa.* The growth of MRSA strains was most effectively inhibited by the bioactive secondary metabolites extracted of *P. aeruginosa* SD01. When exposed to MIC concentration of values of the test compounds that is, 32 µg/mL and 128 µg/mL, *S. aureus* ATCC 25,923 and MRSA ATCC 33,592 cells formed irregular clusters in the SEM investigation. In agreement with other studies, together, these findings suggest that the ethyl acetate fraction may contain special antibacterial molecules that alter cellular metabolism and impair cell division through membrane-targeted pore formation and membrane disruption, which infrequently cause bacterial drug resistance^36,37^. Our findings suggest that since the test fraction had strong anti-staphylococcal and anti-MRSA activity by probably acting on the cell surface, it could present a low risk of developing drug resistance, and therefore this fraction might be thought as an alternative to traditional antibiotics, especially those used for the treatment of skin and mucosal infections.

In this investigation, the crude bioactive secondary metabolite of SD01 exhibited significant anticancer activity against the two cancer cell lines used in the study. Concerning the anticancer screening, it was conducted on the MCF-7 human breast adenocarcinoma, A549 lung carcinoma cell lines. Very promising results were found for the cytotoxic profile of the crude extract from SD01. Compounds produced by this strain have the capacity to inhibit 84%, and 72% of the growth of MCF7, A549 cancer cell lines, respectively. The significant cytotoxic response of this strain against MCF7 and A549 suggests that the extract of this strain may include some powerful anticancer compounds. Several studies have previously demonstrated the cytotoxic activities of marine endophytic extracts in cancer cells^38–40^. These results are consistent with prior studies suggesting that *Pseudomonas* species have the ability to generate anticancer agents that can combat various types of human cancer cells^41,42^. Despite our findings, certain studies have shown that the secondary metabolite produced by Pseudomonas has no significant effect on UMG87 glioblastoma cells and A549 lung carcinoma cells^43^.

It has been observed that natural products provide apoptosis-modulating templates, which can be extremely beneficial in management and therapy of cancer. Hence, it is imperative that apoptotic inducers, either in the form of crude extracts or as isolated compounds, be screened thoroughly^40^. In this investigation, according to flow cytometry analysis, MCF7 and A549 cancer cell lines treated with SD01 bioactive secondary metabolite at the IC50 concentration (100 µg/ml) showed 58% and 64% of cells in the apoptosis stage, respectively. Similar to our study, multiple cancer cell lines have demonstrated apoptosis-mediated cell death in response to bioactive secondary metabolites derived from endophytic organisms of varied plant hosts and related hosts^40^.

The most important of bioactive secondary metabolites identified in this study were, 2-aminoacetophenone, Pyrrolo (1,2-a) pyrazine-1,4-dione, Phenazine, 2-Phenyl-4-cyanopyridine. 2-aminoacetophenone is a volatile molecule with a single benzene ring with a grape-like odor of *P. aeruginosa* cultures^44^. It has been shown that this effector molecule can promote persistent phenotypes via its effects on both the bacterial cell and the host, leading to the long-term bacterial survival at a stationary phase and a reduction in bacterial virulence in various hosts^45^. Regarding its role in relation to plants, it probably aids in the survival of bacteria inside the plant tissue as well as protects the bacteria from the plant's defenses. There is evidence that benzene-1,2-dicarboxylic acid play a role in antimicrobial activity^46^. It has been shown that cyanopyridine with a carbon–nitrogen bond has significant anticancer properties^47^. Phenazines, one of the most widely used bacterial secondary metabolites, have broad-spectrum antibiotic properties against a wide range of bacteria and fungi pathogens. *Pseudomonas* and *Streptomyces* are among the most common species of bacteria that produce phenazine compounds^48^.

## Conclusion

The bacterial endophytes are widely recognized for their innate ability to produce bioactive metabolites with therapeutic applications. Our research offers a glimpse into the antibacterial and anticancer characteristics of these bioactive secondary substances derived from bacterial endophytes linked to the medicinal plant *A. absinthium*. Our findings with regard to the antibacterial and anticancer tests demonstrate the promising utility of endophytic bacteria recovered from medicinal plants in isolating pure bioactive compounds and investigating potential drug breakthroughs. Further investigation needs to be carried out for extraction of novel bioactive chemical compounds recovered from previously known and unknown microorganisms of vast untapped resources that flourish in the arid climate of Iran.

## Materials and methods

### Isolation and Identification of bacterial endophytes

#### Sample collection and isolation of bacterial endophytes

Fresh and viable plant samples from the Isfahan province located in central Iran (32° 27′ 46.0″ N 51° 49′ 37.9″ E) collected employing a randomized sampling technique. We were given permission to collect *A. absinthium* after receiving consent from the university's ethical committee and the Isfahan Agricultural and Natural Resources Research and Education Center. Each sample was identified based on its morphological characteristics. An herbarium specimen (voucher no. ESN12564) provided by Isfahan Agricultural and Natural Resources Research and Education Center used as a botanical reference material to identify and distinguish the collected plants from one another. The plant samples were kept in plastic bags and stored at 4 °C in a refrigerator before isolation. The bacterial isolation procedures were conducted with meticulous attention to sterility. In brief, plant samples were cleaned with distilled water twice to remove the attached microbes and particles on plant surfaces. The samples underwent surface sterilization through immersion in a 70% (v/v) ethanol solution for one minute, followed by treatment with a 5% (v/v) sodium hypochlorite solution (NaOCl) for a period of five minutes. Subsequent to this, the samples were thoroughly rinsed five times with sterile distilled water in order to ensure complete sterilization^49^. The surface sterilized samples were dried on sterile filter paper, cut into small pieces, and were well homogenized in the phosphate-buffered saline (PBS) buffer. Subsequent to this, a homogenized suspension of 100 µl was subjected to culturing on TSA and subjected to incubation at a temperature of 28 °C. Periodic monitoring was conducted over the course of a 5-day incubation period to track growth. The effectiveness of sterilization was evaluated by examining the practicality of the wash control plate.

Obtaining pure cultures was accomplished by selecting distinct colonies and subsequently sub-culturing them on the suitable tryptic soy agar (TSA) medium. The pure bacterial isolates were maintained by employing a preservation technique involving 30% glycerol that was added at a ratio of 1 mL glycerol to 1 mL of overnight broth culture. These samples were subsequently conserved at a temperature of − 80 °C.

#### Biochemical and Molecular Identification of Endophytic Bacteria

The identification of the endophytic bacterial isolates was accomplished through the implementation of a series of methodologies consisting of Gram staining, spore formation analysis, motility assessment, colony pigmentation observation, and meticulous execution of relevant biochemical tests. The strains were identified to species level through the utilization of 16S rRNA gene sequences. In brief, DNA isolation was performed by the modified method of^50^, and 16S rDNA gene was amplified in polymerase chain reaction (PCR) using the genomic DNA as template and bacterial universal primers, 27F (5′-GAGTTTGAT CACTGGCTCAG-3′) and 1492 R (5′-TACGGC TACCTTGTTACGACTT-3′). The PCR products were sequenced by Sanger sequencing method. The sequence of the PCR product was compared with known 16S rRNA gene sequences in the GenBank database by multiple sequence alignment using the MEGA software package version 11^51^ (https://www.megasoftware.net/). The gene sequences were then submitted to the Genbank database and assigned an accession number.

### Bioactivity evaluation

#### Fermentation and extraction

Bioactive secondary metabolites were extracted from the endophytic bacteria using a modified method described by Sebol et al.^34^. The pure endophytic bacteria isolated from *A. absinthium* were cultured in 250 mL culture flasks containing 100 mL of the TSB medium on a rotary shaker at 28 °C for five days at 200 rpm. The cells were then collected by centrifugation at 12,000 rpm at 4 °C for 20 min. The Metabolite was extracted by solvent extraction procedure using ethyl acetate as organic solvent. An equal volume (1:1) an equal volume of ethyl acetate was added to the filtrate containing, and subjected to thorough mixing for a duration of 60 min, followed by a stationary phase ranging between 15 and 30 min to facilitate the separation of the aqueous and organic phases. The fermented broth was filtered and evaporated under vacuum to obtain crude extract. The final concentrated extracts were transferred to pre‒weighed tubes and dried at room temperature for at least 24 h and the dried residues were subjected to thin layer chromatographic (TLC) analysis for partial separation of active metabolites. The crude dried metabolites were re-dissolved in ethyl acetate and methanol (9:1) at a concentration of 20 mg/ml and 10 μl of solution was spotted on TLC plates. Retention factor (Rf) values for all the bands were calculated under ultraviolet light. The compounds related to each band were collected by scratching and dissolving in one mL of ethyl acetate. The ethyl acetate portion was separated by centrifugation at 6000 rpm for 10 min and was evaporated to dryness. The dried residues were placed in a glass vial and stored at − 20 °C for further experiments. Biological assays were conducted after dilutions of partial separation of active metabolites with 10% dimethyl sulfoxide (DMSO) and sterilized by filtration using a PTFE Millipore filter (0.22 µm).

#### Screening for antibacterial activity

Antimicrobial activity of the bioactive secondary metabolites was studied by agar well diffusion method against *Staphylococcus aureus* ATCC 25,923, Methicillin-resistant *S. aureus* ATCC 33,592, *Enterococcus faecium* ATCC 51,299, *Salmonella enterica* ATCC 14,028, *Pseudomonas aeruginosa* (*P. aeruginosa*) 27,852, *Acinetobacter baumannii* ATCC 19,606 at the concertation 10 mg/mL. Antimicrobial activity was detected by measuring the zone of inhibition appeared after the incubation period. A concentration of 10% DMSO was used as a negative control, while cefoxitin, oxacillin, and gentamicin antibiotic discs were used as positive controls.

#### Determination of minimum inhibitory concentration

For the bioactive secondary metabolites with the zone of inhibition larger than 5mm, the MIC was determined by broth microdilution assay as described in the CLSI standard guidelines^52^. Briefly, concentrated crude bioactive secondary metabolite extracts were serially diluted (2048–15.625 μg/mL) in Muller‒Hinton broth and distributed in triplicate in 96-well plates at a final volume of 100 μl. Inoculations comprised of 5 × 10^5^ colony-forming units per milliliter of pathogenic bacteria were introduced into each well, followed by homogenization through mixing. The resulting mixture was then subjected to incubation at 37 °C for a duration of 18 h. Uncultured growth media was used as negative control., and meropenem and vancomycin were used as positive controls.

#### Scanning electron microscopic studies

Scanning electron microscopic** (**SEM) was used to determine the effectiveness of the crude bioactive secondary metabolite extracted from *P. aeruginosa* SD01 after it demonstrated strong antimicrobial activity against *S. aureus* and MRSA strains. Both treated and untreated bacterial cells were harvested by centrifugation at 3000 rpm for ten minutes. In PBS containing 2% glutaraldehyde (v/v), bacterial cell suspensions were directly pre-fixed for 10 min at room temperature. A drop of the fixed cells was deposited onto an aluminum slide with a 10-mm diameter and placed into a polystyrene plate with 6 wells., Bacteria were allowed to settle at room temperature for two hours. The samples were carefully immersed in PBS containing 2 percent (v/v) glutaraldehyde for 10 min at room temperature. The bacterial cells were dehydrated using a range of alcohol concentrations (30 -100 percent), holding them for 10 min in each grade. The dehydrated cells were coated with gold using an ion sputter (Coater IB-2, Gikeengeering, Japan) and finally observed under scanning electron microscope (Zeiss) following the standard method.

#### Anticancer assays by MTT method

Two ATCC cancer cell lines i.e., MCF-7 human adenocarcinoma (breast cancer) and A549 lung carcinoma (Pasteur Institute, Tehran, Iran), were tested for anticancer activity of crude bioactive secondary metabolites from bacterial endophytes. Doxorubicin was used as the positive control. Additionally, L929 normal fibroblast cell line was used to evaluate cytotoxicity of crude bioactive secondary metabolites. Briefly, 100 μl of cell suspension (1 × 10^4^ cells/well) in RPMI 1640 medium supplemented with 10% fetal bovine serum (FBS), 100 U/ml of penicillin, and 100 µg/ml of streptomycin, as seeded in 96-well microplates and incubated for 24 h at 37 °C, 5% CO_2_, and 98% air humidified. The old media were discarded and 100 μl of the prepared different concentrations of crude bioactive secondary metabolites were added to each well. The plates were incubated for 24 h in the same condition. Doxorubicin and DMSO 1% were used as positive and negative controls, respectively. Cell survival was determined by adding 10 µl of MTT solution (5 mg/mL) to each well and incubating the plates at 37 °C for 3 h. The old MTT-containing media was gently removed, and 100 µl DMSO was pipetted into each well to dissolve the formed formazan crystals. At 570 nm, absorbance was measured using an ELISA plate reader (Startfix-2100, Awareness, USA). The concentrations that inhibit half of the cell population (IC_50_) were obtained by modeling the percentage of cytotoxicity versus concentration of crude bioactive secondary metabolites. The results are presented as mean ± standard deviation (SD).

#### Apoptosis evaluation by an Annexin V binding assay

The Annexin V binding assay was used to identify the changes to the plasma membrane that occur during the early stages of the apoptotic cell death process using FITC Annexin V Apoptosis Detection Kit, BioLegend (San Diego, California) according to the manufacturer's instructions., MCF-7 human adenocarcinoma and A549 lung carcinoma cells (1 × 10^6^ cell/well) were treated with crude bioactive secondary metabolites (1 mg/ml) and incubated at 37 °C, 5% CO2 for 24 h. The supernatant in each well was carefully removed, and the cells were washed with PBS. The harvested cells were then resuspended in 400 µl of Annexin V binding buffer containing 2 µl of 0.5 mg/mL PI and 5 µl of Annexin V-FITC. The cells were incubated for 15 min at room temperature in the dark. The cells were analyzed after incubation using a BD FACSCalibur flow cytometer (Biosciences, San Jose, CA, USA). The cells were differentiated into four quadrates based on the cell stages: viable (Q4), early apoptosis (Q3), late apoptosis (Q2), and necrosis (Q1) quadrates. The flow cytometry analysis was conducted using FlowJo software (version 10.5.3, TreeStar Incorporated, Ashland, OR).

### Identification of bioactive secondary metabolites

#### FTIR Spectrophotometer analysis

The crude bioactive secondary metabolite extracted from *P. aeruginosa* SD01 by TLC were analyzed by FTIR to determine the various functional groups present in bioactive secondary metabolites. using a Shimadzu 8400S spectrophotometer (Shimadzu Corporation, Japan) with a wavelength range of 400–4000 cm^-1^. The dried bioactive secondary metabolite was mixed with potassium bromide (KBr) to form a very fine powder. A thin pellet was then made from this powder and analyzed. Infrared light was also transparent to KBr. The graph was used to interpret various functional groups.

#### Gas Chromatography mass spectrometry (GC–MS) analysis

The crude bioactive secondary metabolite extracted from *P. aeruginosa* SD01 was subjected to GC–MS analysis to identify the bioactive compounds. The sample was analyzed in Hewlett-Packard 5890 gas chromatograph equipped with HP 5972 MSD detector. An SLB5 column DB-5 ms (30 m, 0.25 mm film thickness) was used with a 30 min temperature program of 60–320 at 10 °C /min followed by a 5 min hold at 320 °C. The injector temperature was 250 °C; the flow rate of the carrier gas (helium) was 1 mL/min; and the split ratio was 1:50. The interval of the scan m/z was between 35 and 900 and the identification of the compounds was based on an individual spectrum comparison of each compound in the Wiley07 and NIST08 library data. The retention index was calculated using the mixture of alkanes standard from C10 to C40 (Merck KGaA, Darmstadt, Germany).

### Ethics approval

All phases of the study have received ethical approval from the ethics committee of Isfahan University of Medical Sciences, Isfahan, Iran (approval number: IR.MUI.MED.REC.1398.605). https://ethics.research.ac.ir/ProposalCertificateEn.php?id=119531&Print=true&NoPrintHeader=true&NoPrintFooter=true&NoPrintPageBorder=true&LetterPrint=true.

### Statement confirming of experimental research and field studies on plants

We certify that this study complies with relevant institutional, national, and international guidelines and legislation of experimental research and field studies on plants (either cultivated or wild), including the collection of plant material.

### Statement of identified the plant

The plant used in this study was formal identified by Dr. Azadeh Akhavan of the Isfahan Agricultural and Natural Resources Research, in Isfahan, Iran. A voucher specimen of the plant material has not been deposited in a publicly accessible herbarium because the Isfahan Agricultural and Natural Resources Research Center is a specialized scientific center for identifying plants and its herbarium is not accessible to the public.

## Data Availability

The 16S rRNA encoding gene sequences of the isolated endophytic bacteria from *A. absinthium* were deposited in the GenBank database under accession numbers ON954839, ON965228, ON965226, ON965227, ON965231, ON965230, ON965229, ON965232, ON994625 and ON994626.
